# Entanglement criterion independent on observables for multipartite Gaussian states based on uncertainty principle

**DOI:** 10.1038/s41598-018-36307-1

**Published:** 2019-02-04

**Authors:** Kan He, Miaomiao Huang, Jinchuan Hou

**Affiliations:** College of Mathematics, Institute of Mathematics, Taiyuan University of Technology, Taiyuan, 030024 P. R. China

## Abstract

Quantum entanglement is one of important resources for quantum communication. Entanglement criteria help us detect entangled states. One of important criteria is the local uncertainty relation (LUR) entanglement criteria, which is studied extensively. However, all existent LUR criteria are dependent on the chosen observables. In the paper, applying the uncertainty principle, we improve the LUR criteria to obtain entanglement criteria for multipartite Gaussian states, which are independent on observalbes.

## Introduction

Entanglement, as an important resource in quantum communication, has been focused on extensively in both finite dimensional and infinite dimensional (esp. continuous variable) quantum systems^1–4^. It is one of core problems to decide whether or not a given quantum state is entangled. As we know, continuous variable (CV) quantum systems are fundamental and important from theoretical and experimental views. In particular, Gaussian states can be theoretically easy to manage and experimentally easy to produce^5^. Recently the topics on entanglement of Gaussian states have been developed rapidly. Some different conditions for entanglement of bipartite Gaussian states are extended from the finite dimensional case, such as, the criterion of the positivity of the partial transpose and additional separability criteria for covariance matrices^6,7^, the computable cross norm (CCN) or realignment criterion^8,9^. The above mentioned criteria are also generalized to the multipartite Gaussian states^10–13^. Furthermore, other techniques are also used to build the entanglement criteria for multi partite Gaussian states^14–16^.

Entanglement criteria based on uncertainty relations have been studied in multi partite continuous variable systems^17–22^. Such a technique is found by Duan, Giedke, Cirac and Zoller^17^ and the so-called local uncertainty relation (LUR) criteria are developed by Hofmann and Takeuchi^18^. Roughly speaking, if one want to determine whether or not a CV state is entangled by LUR, it needs to check whether or not the state violates an inequality dependent on chosen observables and parameters. For example, Loock and Furusawa^21^ improve the LUR and says that: for an *N*-party and *N*-mode CV state *ρ*, *ρ* is separable if for arbitrary scalar *h*_1_, *h*_2_, …, *h*_*N*_, *g*_1_, *g*_2_, …, *g*_*N*_

$${\langle {({\rm{\Delta }}\hat{u})}^{2}\rangle }_{\rho }+{\langle {({\rm{\Delta }}\hat{v})}^{2}\rangle }_{\rho }\ge f({h}_{1},{h}_{2},\ldots ,{h}_{N},{g}_{1},{g}_{2},\ldots ,{g}_{N}),$$

where $\hat{u}={\sum }_{i=1}^{N}\,{h}_{i}{\hat{x}}_{i}$, $\hat{v}={\sum }_{i=1}^{N}\,{g}_{i}{\hat{p}}_{i}$, $({\hat{x}}_{i},{\hat{p}}_{i})$ is the pair of the position and momentum operators in the *i*th mode (party) and *f* is a computable function. The criteria in^21^ are available for *N*-party and *N*-mode CV states (that is, there is only one mode in each party) and dependent on observables $\hat{u}$ and $\hat{v}$. However, all of the above criteria are dependent on the choice of observables. In the present paper, improving the LUR criteria, we devote to building an entanglement criterion of Gaussian states, which is independent on observables. Furthermore, it is mentioned that the criterion in^21^ is available for *N*-party and *N*-mode CV states (that is, there is only one mode in each party). The criterion in the present paper can be executed for *N*-party systems with arbitrary modes in each party (See Theorem 2.1 and Corollary 2.2).

Next let us introduce some definitions and notations about Gaussian states. The characteristic function *χ* of an arbitrary *n*-mode density operator *ρ* is defined as

$${\chi }_{n}(\lambda )={\rm{tr}}(\rho D(\lambda )),$$

where $\lambda \in {{\mathbb{R}}}^{2n}$ and $D(\lambda )={\otimes }_{i=1}^{n}D({\lambda }_{i})={\otimes }_{i=1}^{n}\,\exp ({\lambda }_{i}{\hat{a}}_{i}^{\dagger }-{\lambda }_{i}^{\ast }{\hat{a}}_{i})$ is the *n*-mode Weyl displacement operator^23^. Here, ${\hat{a}}_{i}^{\dagger }$ and ${\hat{a}}_{i}$ are the creation and annihilation operators in the ith mode satisfying the canonical commutation relation

$$[{\hat{a}}_{i},{\hat{a}}_{j}^{\dagger }]={\delta }_{ij}\,{\rm{and}}\,[{\hat{a}}_{i}^{\dagger },{\hat{a}}_{j}^{\dagger }]=[{\hat{a}}_{i},{\hat{a}}_{j}]=0.$$

Let $\rho =\frac{1}{{(2\pi )}^{n}}\,\int \,{\chi }_{n}(\lambda )D(\,-\,\lambda ){d}^{2n}\lambda$. *ρ* is called a Gaussian state if *χ*_*n*_(*λ*) is of the form

$${\chi }_{n}(\lambda )=\exp [i{m}^{T}\lambda -\frac{1}{2}{\lambda }^{T}\gamma \lambda ],$$

where $\gamma \in {M}_{2n}({\mathbb{R}})$ and $m\in {{\mathbb{R}}}^{2n}$ denote the covariance matrix (CM) and the mean of *ρ*, respectively. *γ* fulfills the Robertson-Schrödinger uncertainty relation

$$\gamma -\frac{i}{2}J\ge 0,$$

where $J={\oplus }_{i=1}^{n}\,{J}_{i}$ and $${J}_{i}=(\begin{array}{cc}0 & 1\\ -\,1 & 0\end{array})$$. Let ${\hat{p}}_{i}$ and ${\hat{q}}_{i}$ be the position operator and momentum operator on the ith mode,

$$R={[{R}_{1},{R}_{2},\ldots ,{R}_{2n}]}^{T}={[\sqrt{\omega }{\hat{q}}_{1},\frac{{\hat{p}}_{1}}{\sqrt{\omega }},\sqrt{\omega }{\hat{q}}_{2},\frac{{\hat{p}}_{2}}{\sqrt{\omega }},\ldots ,\sqrt{\omega }{\hat{q}}_{n},\frac{{\hat{p}}_{n}}{\sqrt{\omega }}]}^{T},$$

then the CM of *ρ* can be calculated as follows:

$$\gamma -\frac{i}{2}J=({\rm{tr}}(\rho ({R}_{j}-{m}_{j})\,({R}_{k}-{m}_{k}))),$$

where *m*_*j*_ = tr(*ρR*_*j*_) is the *j*th coordinate of the displacement vector *m*. Note that entanglement of a Gaussian state is independent on its displacement, so we assume that all Gaussian states is with zero displacement in the paper. We also recall that a multipartite quantum state *ρ* on ${H}_{1}\otimes {H}_{2}\otimes \cdots \otimes {H}_{n}$ is fully separable if there exist quantum state ${\rho }_{k}^{(i)}$ on *H*_*i*_ such that $\rho =\int \,P(x){\rho }_{i}^{(1)}\otimes {\rho }_{i}^{(2)}\otimes \cdots \otimes {\rho }_{i}^{(n)}dx$, where *P*(*x*) ≥ 0 and $\int \,P(x)dx=1$.

## Results

### Entanglement criteria for multipartite Gaussian states

Let *H*_1_, *H*_2_, …, *H*_*n*_ be complex separable infinite dimensional Hilbert spaces, there are *s*_*i*_ modes in each *H*_*i*_ for arbitrary integers *s*_*i*_ and *i*. Set

$$\begin{array}{ll}{H}_{1}: & {\hat{x}}_{1}^{(1)},{\hat{x}}_{2}^{(1)},\ldots ,{\hat{x}}_{{s}_{1}}^{(1)}\\  & {\hat{p}}_{1}^{(1)},{\hat{p}}_{2}^{(1)},\ldots ,{\hat{p}}_{{s}_{1}}^{(1)}\\ {H}_{2}: & {\hat{x}}_{1}^{(2)},{\hat{x}}_{2}^{(2)},\ldots ,{\hat{x}}_{{s}_{2}}^{(2)}\\  & {\hat{p}}_{1}^{(2)},{\hat{p}}_{2}^{(2)},\ldots ,{\hat{p}}_{{s}_{2}}^{(2)}\\  & \,\,\,\,\,\vdots \\ {H}_{n}: & {\hat{x}}_{1}^{(n)},{\hat{x}}_{2}^{(n)},\ldots ,{\hat{x}}_{{s}_{n}}^{(n)}\\  & {\hat{p}}_{1}^{(n)},{\hat{p}}_{2}^{(n)},\ldots ,{\hat{p}}_{{s}_{n}}^{(n)}\end{array}$$

where $({\hat{x}}_{j}^{(i)},{\hat{p}}_{j}^{(i)})$ is the pair of the position and momentum operators in the *j*th mode of the *i*th party. Sometime we write $({\hat{q}}_{1},{\hat{q}}_{2},{\hat{q}}_{3},\ldots \ldots ,{\hat{q}}_{2{\sum }_{j}{s}_{j}})=({\hat{x}}_{1}^{(1)},{\hat{p}}_{1}^{(1)},{\hat{x}}_{2}^{(1)},\ldots \ldots ,{\hat{p}}_{{s}_{n}}^{(n)})$. Denote by ${\mathscr{S}}(H)$ the set of all quantum states on *H*. We have the following main result.

#### **Theorem 2**.**1**

*Let* $\rho \in {\mathscr{S}}({H}_{1}\otimes {H}_{2}\cdots \otimes {H}_{n})$ *with covariance matrix γ*, *and the* $(2\,{\sum }_{j}\,{s}_{j})\times (2\,{\sum }_{j}\,{s}_{j})$ *matrix* ${M}_{\rho }=({m}_{ij})=\gamma -\frac{i}{2}J$. *If ρ is fully separable, then for two sets of arbitrary real numbers* $\{{\alpha }_{j}^{(i)}\}$ *and* $\{{\beta }_{j}^{(i)}\}$ (*i* = 1, …, *n and j* = 1, …, *s*_*i*_),

$${{\rm{\Gamma }}}_{{M}_{\rho },\alpha ,\beta }={({\gamma }_{k,l}(\alpha ,\beta ))}_{n\times n}\ge 0.$$

*where*

$$\begin{array}{rcl}{\gamma }_{k,k} & = & \sum _{m,h=1}^{{s}_{k}}\,{\alpha }_{m}^{(k)}{\alpha }_{h}^{(k)}{m}_{2\sum _{j=1}^{k-1}{s}_{j}+2m-1,2\sum _{j=1}^{k-1}{s}_{j}+2h-1}\\  &  & +\,\sum _{m,h=1}^{{s}_{k}}\,{\beta }_{m}^{(k)}{\beta }_{h}^{(k)}{m}_{2\sum _{j=1}^{k-1}{s}_{j}+2m,2\sum _{j=1}^{k-1}{s}_{j}+2h}-\sum _{i=1}^{{s}_{k}}\,{\alpha }_{i}^{(k)}{\beta }_{i}^{(k)};\end{array}$$

$$\begin{array}{rcl}{\gamma }_{c,d} & = & \sum _{m=1}^{{s}_{c}}\,\sum _{h=1}^{{s}_{d}}{\alpha }_{m}^{(c)}{\alpha }_{h}^{(d)}{m}_{2\sum _{j=1}^{c-1}{s}_{j}+2m-1,2\sum _{j=1}^{d-1}{s}_{j}+2h-1}\\  &  & +\,\sum _{m=1}^{{s}_{c}}\sum _{h=1}^{{s}_{d}}{\beta }_{m}^{(c)}{\beta }_{h}^{(d)}{m}_{2\sum _{j=1}^{c-1}{s}_{j}+2m,2\sum _{j=1}^{d-1}{s}_{j}+2h}\,(c\ne d).\end{array}$$

#### **Proof**.

See the method section.

The following example helps us understand how to obtain the matrix ${{\rm{\Gamma }}}_{{M}_{\rho },\alpha ,\beta }$ defined in Theorem 2.1 from *M*_*ρ*_.

Next we will design a optimization program for entanglement criteria of Gaussian states. Firstly, we have the following corollary from Theorem 2.1.

#### **Corollary 2**.**2**

*There exists entanglement among* ${H}_{{i}_{1}},{H}_{{i}_{2}},\ldots ,{H}_{{i}_{m}}$ (*i*_*l*_ ∈ {1, 2, …, *n*}, *i*_*s*_ ≤ *i*_*t*_ if *s* ≤ *t*, *m* ≤ *n*) *if the scalar*

$${\lambda }_{{i}_{1},{i}_{2},\ldots ,{i}_{m}}({M}_{\rho }) < 0,$$

*where*

$${\lambda }_{{i}_{1},{i}_{2},\ldots ,{i}_{m}}({M}_{\rho })=\mathop{{\rm{\min }}}\limits_{1\le l\le m}\,\mathop{{\rm{\min }}}\limits_{{i}_{1}\le k\le {i}_{l}}\,\mathop{{\rm{\min }}}\limits_{\{{\alpha }_{j}^{(i)}\},\{{\beta }_{j}^{(i)}\}}\,|{{\rm{\Gamma }}}_{k}({i}_{1},{i}_{2},\ldots ,{i}_{l})|,$$

|Γ_*k*_(*i*_1_, *i*_2_, …, *i*_*l*_)| *is the kth leading principal minor of the submatrix* Γ(*i*_1_, *i*_2_, …, *i*_*l*_) *of* $${{\rm{\Gamma }}}_{{M}_{\rho },\alpha ,\beta }$$, Γ(*i*_1_, *i*_2_, …, *i*_*l*_) *is obtained by removing the sth row and the sth column of* $${{\rm{\Gamma }}}_{{M}_{\rho },\alpha ,\beta }$$ *for all s* ∈ {1, 2, …, *n*}\{*i*_1_, *i*_2_, …, *i*_*l*_}.

Applying the Corollary 2.2, we can detect entanglement of a multi-party Gaussian state by solving the following optimization problem.

Let $\rho \in {\mathscr{S}}({H}_{1}\otimes {H}_{2}\cdots \otimes {H}_{n})$ with ${M}_{\rho }={({m}_{ij})}_{(2{\sum }_{j}{s}_{j})\times (2\sum {s}_{j})}$. To detect whether or not there exists entanglement among the given parts ${H}_{{i}_{1}},{H}_{{i}_{2}},\ldots ,{H}_{{i}_{m}}$, it is the key to minimize |Γ_*k*_(*i*_1_, *i*_2_, …, *i*_*l*_)| in Corollary 2.2 for any fixed *l*, *k*.

OP $$\begin{array}{ll}{\rm{Minimize}}\,: & |{{\rm{\Gamma }}}_{k}({i}_{1},{i}_{2},\ldots ,{i}_{l})|\\ {\rm{Subject}}\,{\rm{to}}\,: & \{{\alpha }_{j}^{(i)}\}\subseteq {\mathbb{R}},\{{\beta }_{j}^{(i)}\}\subseteq {\mathbb{R}},(i=1,\ldots ,n,j=1,\ldots ,{s}_{i})\end{array}$$

We design the following steps to solve the OP problem.

S1. We compute and collect leading principal minors |Γ_*k*_(*i*_1_, *i*_2_, …, *i*_*l*_)|, it is a polynomial $p(\{{\alpha }_{j}^{({i}_{k})}\},\{{\beta }_{j}^{({i}_{k})}\})$ with $2\,{\sum }_{t=1}^{k}\,{s}_{{i}_{t}}$ variables $\{{\alpha }_{j}^{({i}_{k})}\},\{{\beta }_{j}^{({i}_{k})}\}$;

S2. We compute partial derivative $\partial p/\partial {\alpha }_{j}^{({i}_{k})}$ and $\partial p/\partial {\beta }_{j}^{({i}_{k})}$ of $p(\{{\alpha }_{j}^{({i}_{k})}\},\{{\beta }_{j}^{({i}_{k})}\})$ for each variable respectively;

S3. We get stationary points of the equation set consist of $\partial p/{\beta }_{j}^{({i}_{k})}=\partial p/{\beta }_{j}^{({i}_{k})}=0$;

S4. We compute the local minimal values of polynomial $p(\{{\alpha }_{j}^{({i}_{k})}\},\{{\beta }_{j}^{({i}_{k})}\})$ on all stationary points. Finally we obtain the minimal value of |Γ_*k*_(*i*_1_, *i*_2_, …, *i*_*l*_)|.

Now, we consider an example of the multi-mode pure symmetric Gaussian state is introduced in^24^. Arbitrary a 5-mode pure symmetric Gaussian state *ρ*_*s*_ has the following covariance matrix:

1 $${\gamma }_{s}=(\begin{array}{llllllllll}a & 0 & {c}_{1} & 0 & {c}_{1} & 0 & {c}_{1} & 0 & {c}_{1} & 0\\ 0 & a & 0 & {c}_{2} & 0 & {c}_{2} & 0 & {c}_{2} & 0 & {c}_{2}\\ {c}_{1} & 0 & a & 0 & {c}_{1} & 0 & {c}_{1} & 0 & {c}_{1} & 0\\ 0 & {c}_{2} & 0 & a & 0 & {c}_{2} & 0 & {c}_{2} & 0 & {c}_{2}\\ {c}_{1} & 0 & {c}_{1} & 0 & a & 0 & {c}_{1} & 0 & {c}_{1} & 0\\ 0 & {c}_{2} & 0 & {c}_{2} & 0 & a & 0 & {c}_{2} & 0 & {c}_{2}\\ {c}_{1} & 0 & {c}_{1} & 0 & {c}_{1} & 0 & a & 0 & {c}_{1} & 0\\ 0 & {c}_{2} & 0 & {c}_{2} & 0 & {c}_{2} & 0 & a & 0 & {c}_{2}\\ {c}_{1} & 0 & {c}_{1} & 0 & {c}_{1} & 0 & {c}_{1} & 0 & a & 0\\ 0 & {c}_{2} & 0 & {c}_{2} & 0 & {c}_{2} & 0 & {c}_{2} & 0 & a\end{array})$$

where *a* ≥ 1 and

$${c}_{1}=\tfrac{3({a}^{2}-1)+\sqrt{({a}^{2}-1)\,(25{a}^{2}-9)}}{8a},\,{c}_{2}=\tfrac{3({a}^{2}-1)-\sqrt{({a}^{2}-1)\,(25{a}^{2}-9)}}{8a}.$$

In^24^, entanglement of the above state in the case *a* = 1.2 is discussed^16^. Here we take *a* = 1.1.

We first deal with the partition 1|2|3|4|5, that is, five modes and five parties. In order to determine when the state *ρ*_*s*_ with the covariance matrix in Eq. (1) is entangled, it follows from Theorem 2.1 and Corollary 2.2 that we need to check when the following matrix Γ_*s*_, which is restructured by ${M}_{\rho }={\gamma }_{s}-\frac{i}{2}J$, is not positive for some real scalars *α*_*i*_ and *β*_*i*_, *i* = 1, 2, 3, 4, 5

2 $${{\rm{\Gamma }}}_{s}=(\begin{array}{ccccc}a({\alpha }_{1}^{2}+{\beta }_{1}^{2})-{\alpha }_{1}{\beta }_{1} & {c}_{1}{\alpha }_{1}{\alpha }_{2}+{c}_{2}{\beta }_{1}{\beta }_{2} & {c}_{1}{\alpha }_{1}{\alpha }_{3}+{c}_{2}{\beta }_{1}{\beta }_{3} & {c}_{1}{\alpha }_{1}{\alpha }_{4}+{c}_{2}{\beta }_{1}{\beta }_{4} & {c}_{1}{\alpha }_{1}{\alpha }_{5}+{c}_{2}{\beta }_{1}{\beta }_{5}\\ {c}_{1}{\alpha }_{1}{\alpha }_{2}+{c}_{2}{\beta }_{1}{\beta }_{2} & a({\alpha }_{2}^{2}+{\beta }_{2}^{2})-{\alpha }_{2}{\beta }_{2} & {c}_{1}{\alpha }_{2}{\alpha }_{3}+{c}_{2}{\beta }_{2}{\beta }_{3} & {c}_{1}{\alpha }_{2}{\alpha }_{4}+{c}_{2}{\beta }_{2}{\beta }_{4} & {c}_{1}{\alpha }_{2}{\alpha }_{5}+{c}_{2}{\beta }_{2}{\beta }_{5}\\ {c}_{1}{\alpha }_{1}{\alpha }_{3}+{c}_{2}{\beta }_{1}{\beta }_{3} & {c}_{1}{\alpha }_{2}{\alpha }_{3}+{c}_{2}{\beta }_{2}{\beta }_{3} & a({\alpha }_{3}^{2}+{\beta }_{3}^{2})-{\alpha }_{3}{\beta }_{3} & {c}_{1}{\alpha }_{3}{\alpha }_{4}+{c}_{2}{\beta }_{3}{\beta }_{4} & {c}_{1}{\alpha }_{3}{\alpha }_{5}+{c}_{2}{\beta }_{3}{\beta }_{5}\\ {c}_{1}{\alpha }_{1}{\alpha }_{4}+{c}_{2}{\beta }_{1}{\beta }_{4} & {c}_{1}{\alpha }_{2}{\alpha }_{4}+{c}_{2}{\beta }_{2}{\beta }_{4} & {c}_{1}{\alpha }_{3}{\alpha }_{4}+{c}_{2}{\beta }_{3}{\beta }_{4} & a({\alpha }_{4}^{2}+{\beta }_{4}^{2})-{\alpha }_{4}{\beta }_{4} & {c}_{1}{\alpha }_{4}{\alpha }_{5}+{c}_{2}{\beta }_{4}{\beta }_{5}\\ {c}_{1}{\alpha }_{1}{\alpha }_{5}+{c}_{2}{\beta }_{1}{\beta }_{5} & {c}_{1}{\alpha }_{2}{\alpha }_{5}+{c}_{2}{\beta }_{2}{\beta }_{5} & {c}_{1}{\alpha }_{3}{\alpha }_{5}+{c}_{2}{\beta }_{3}{\beta }_{5} & {c}_{1}{\alpha }_{4}{\alpha }_{5}+{c}_{2}{\beta }_{4}{\beta }_{5} & a({\alpha }_{5}^{2}+{\beta }_{5}^{2})-{\alpha }_{5}{\beta }_{5}\end{array}).$$

When *a* = 1.1, we calculate and obtain that the minimal values of 2 × 2 five leading principal minors of Γ_*s*_ are all negative by Mathematica. So Γ_*s*_ is not positive. It follows that the corresponding symmetric Gaussian state is entangled in the partition 1|2|3|4|5.

Furthermore, now if one want to ask whether or not there exists entanglement among the second, fourth and fifth mode. Then we only need to check positivity of the following Γ_*s*_:

3 $$(\begin{array}{ccc}a({\alpha }_{2}^{2}+{\beta }_{2}^{2})-{\alpha }_{2}{\beta }_{2} & {c}_{1}{\alpha }_{2}{\alpha }_{4}+{c}_{2}{\beta }_{2}{\beta }_{4} & {c}_{1}{\alpha }_{2}{\alpha }_{5}+{c}_{2}{\beta }_{2}{\beta }_{5}\\ {c}_{1}{\alpha }_{2}{\alpha }_{4}+{c}_{2}{\beta }_{2}{\beta }_{4} & a({\alpha }_{4}^{2}+{\beta }_{4}^{2})-{\alpha }_{4}{\beta }_{4} & {c}_{1}{\alpha }_{4}{\alpha }_{5}+{c}_{2}{\beta }_{4}{\beta }_{5}\\ {c}_{1}{\alpha }_{2}{\alpha }_{5}+{c}_{2}{\beta }_{2}{\beta }_{5} & {c}_{1}{\alpha }_{4}{\alpha }_{5}+{c}_{2}{\beta }_{4}{\beta }_{5} & a({\alpha }_{5}^{2}+{\beta }_{5}^{2})-{\alpha }_{5}{\beta }_{5}\end{array}).$$

When *a* = 1.1, the matrix (3) is not positive, and so there exists entanglement between the second, fourth and fifth mode, similar to the discussion of the case of partition 1|2|3|4|5.

## Discussion

The local uncertainty relation (LUR) criterion is one of important classes of entanglement criteria for the continuous variable system. It is dependent on chosen observables. Here, we improve LUR criteria and obtain observable-independent entanglement criteria for arbitrary multi-party and multi-mode Gaussian states. In particular, the criteria can be implemented by a by a minimum optimization computer program. It is also mentioned that one of the further open problems is to discuss the computational complexity of the optimization procedure of the OP problem.

## Methods

Before the proof of Theorem 2.1, we need the following lemmas. The following lemma can be checked straightforwardly.

### **Lemma 1**.

*Let*

$$\begin{array}{rcl}{\hat{X}}^{(k)} & = & \sum _{i=1}^{{s}_{k}}\,{\alpha }_{i}^{(k)}{\hat{x}}_{i}^{(k)}\\ {\hat{P}}^{(k)} & = & \sum _{i=1}^{{s}_{k}}\,{\beta }_{i}^{(k)}{\hat{p}}_{i}^{(k)}\end{array}$$

*Then*

$$[{\hat{X}}^{(k)},{\hat{P}}^{(k)}]=i(\sum _{i=1}^{{s}_{k}}{\alpha }_{i}^{(k)}{\beta }_{i}^{(k)})I;$$

$$[{\hat{X}}^{(k)},{\hat{P}}^{(m)}]=0,(k\ne m);$$

$${\hat{X}}^{(k)}{\hat{X}}^{(m)}={\hat{X}}^{(m)}{\hat{X}}^{(k)};$$

$${\hat{P}}^{(k)}{\hat{P}}^{(m)}={\hat{P}}^{(m)}{\hat{P}}^{(k)}.$$

### **Lemma 2**.

*Let* $$\begin{array}{c}{\{{t}_{i}\}}_{i=1}^{n}\end{array}$$ *be a set of arbitrary real numbers*. *Let*

$$\begin{array}{rcl}U & = & \sum _{k=1}^{n}\,{t}_{k}\,{\hat{X}}^{(k)}\\ V & = & \sum _{k=1}^{n}\,{t}_{k}{\hat{P}}^{(k)}\end{array}$$

*and* $\rho \in S({H}_{1}\otimes {H}_{2}\otimes \cdots \otimes {H}_{n})$. *If ρ is fully separable*, *then*

$${({\rm{\Delta }}U)}^{2}+{({\rm{\Delta }}V)}^{2}\ge \sum _{k=1}^{n}\,(\sum _{i=1}^{{s}_{k}}\,{\alpha }_{i}^{(k)}{\beta }_{i}^{(k)}){t}_{k}^{2}.$$

### *Proof*.

Since *ρ* is fully separable,

$$\rho =\int \,P(x){\rho }_{i}^{(1)}\otimes {\rho }_{i}^{(2)}\otimes \cdots \otimes {\rho }_{i}^{(n)}dx.$$

Writing ${\langle A\rangle }_{i}={\rm{tr}}(A{\rho }_{i}^{(1)}\otimes {\rho }_{i}^{(2)}\otimes \cdots \otimes {\rho }_{i}^{(n)})$, and as we know that entanglement of Gaussian states is independent on the mean, we have

$$\begin{array}{rcl}{({\rm{\Delta }}U)}^{2}+{({\rm{\Delta }}V)}^{2} & = & \int P(x)dx[\sum _{k=1}^{n}({t}_{k}^{2}\langle {({\hat{X}}^{(k)})}^{2}{\rangle }_{i}+{t}_{k}^{2}{\langle {({\hat{P}}^{(k)})}^{2}\rangle }_{i})\\  &  & +\,\sum _{l < j}2{t}_{l}{t}_{j}{\langle {\hat{X}}^{(l)}\rangle }_{i}{\langle {\hat{X}}^{(j)}\rangle }_{i}+\,\sum _{l < j}2{t}_{l}{t}_{j}{\langle {\hat{P}}^{(l)}\rangle }_{i}{\langle {\hat{P}}^{(j)}\rangle }_{i}]-\,{\langle U\rangle }_{\rho }^{2}-{\langle V\rangle }_{\rho }^{2}\\  & = & \int P(x)dx[\sum _{k=1}^{n}({t}_{k}^{2}{\langle {({\hat{X}}^{(k)})}^{2}\rangle }_{i}+{t}_{k}^{2}{\langle {({\hat{P}}^{(k)})}^{2}\rangle }_{i})\\  &  & +\,\sum _{l < j}2{t}_{l}{t}_{j}{\langle {\hat{X}}^{(l)}\rangle }_{i}{\langle {\hat{X}}^{(j)}\rangle }_{i}+\,\sum _{l < j}2{t}_{l}{t}_{j}{\langle {\hat{P}}^{(l)}\rangle }_{i}{\langle {\hat{P}}^{(j)}\rangle }_{i}]-\,{\langle U\rangle }_{\rho }^{2}-{\langle V\rangle }_{\rho }^{2}\\  &  & -\,\int P(x)dx[\sum _{k=1}^{n}({t}_{k}^{2}\langle {\hat{X}}^{(k)}{\rangle }_{i}^{2}+{t}_{k}^{2}{\langle {\hat{P}}^{(k)}\rangle }_{i}^{2})]\\  &  & +\,\int P(x)dx[\sum _{k=1}^{n}({t}_{k}^{2}\langle {\hat{X}}^{(k)}{\rangle }_{i}^{2}+{t}_{k}^{2}{\langle {\hat{P}}^{(k)}\rangle }_{i}^{2})]\\  & = & \int P(x)dx[\sum _{k=1}^{n}({t}_{k}^{2}{({\rm{\Delta }}{\hat{X}}^{(k)})}_{i}^{2}+{t}_{k}^{2}{({\rm{\Delta }}{\hat{P}}^{(k)})}_{i}^{2})]\\  &  & +\,\int P(x)dx[{(\sum _{k=1}^{n}{t}_{k}{\langle {\hat{X}}^{(k)}\rangle }_{i})}^{2}+{(\sum _{k=1}^{n}{t}_{k}{\langle {\hat{P}}^{(k)}\rangle }_{i})}^{2}]-\,{\langle U\rangle }_{\rho }^{2}-{\langle V\rangle }_{\rho }^{2}\\  & \ge  & \sum _{k=1}^{n}{t}_{k}^{2}\langle [{\hat{X}}^{(k)},{\hat{P}}^{(k)}]\rangle =\sum _{k=1}^{n}(\sum _{k=1}^{{s}_{k}}{\alpha }_{i}^{(k)}{\beta }_{i}^{(k)}){t}_{k}^{2}\end{array}$$

$$\square $$

### Proof of Theorem 2.1

On the one hand, it follows from Lemma 0.2 that

$${\rm{\Delta }}{U}^{2}+{\rm{\Delta }}{V}^{2}\ge \sum _{k-1}^{n}\,(\sum _{i\mathrm{=1}}^{{s}_{k}}\,{\alpha }_{i}^{k}{\beta }_{i}^{k}){t}_{k}^{2}\mathrm{.}$$

Note that entanglement of a Gaussian state is independent on its first moment (i.e., the mean) of a Gaussian state, so we assume that 〈*U*〉 = 0 = 〈*V*〉. On the other hand,

$$\begin{array}{c}{\rm{\Delta }}{U}^{2}+{\rm{\Delta }}{V}^{2}\\ \begin{array}{ll}= & \langle {U}^{2}\rangle -{\langle U\rangle }^{2}+\langle {V}^{2}\rangle -{\langle V\rangle }^{2}\\ = & {\langle {[{\sum }_{i=1}^{n}{t}_{i}({\sum }_{j=1}^{{s}_{i}}{\alpha }_{j}^{(i)}{\hat{x}}_{j}^{(i)})]}^{2}\rangle }_{\rho }+\,\,{\langle {[{\sum }_{i=1}^{n}{t}_{i}({\sum }_{j=1}^{{s}_{i}}{\beta }_{j}^{(i)}{\hat{p}}_{j}^{(i)})]}^{2}\rangle }_{\rho }-{\langle U\rangle }^{2}-{\langle V\rangle }^{2}\\ = & {\sum }_{i=1}^{n}\,{t}_{i}^{2}{\rm{tr}}[({\sum }_{m,h=1}^{{s}_{i}}\,{\alpha }_{m}^{(i)}{\alpha }_{h}^{(i)}{\hat{x}}_{m}^{(i)}{\hat{x}}_{h}^{(i)})\rho ]\\  & +\,{\sum }_{i\ne j}^{n}\,{t}_{i}{t}_{j}{\rm{tr}}[({\sum }_{m=1}^{{s}_{i}}{\sum }_{h=1}^{{s}_{j}}\,{\alpha }_{m}^{(j)}{\alpha }_{h}^{(j)}{\hat{x}}_{m}^{(j)}{\hat{x}}_{h}^{(j)})\rho ]\,\\  & +\,{\sum }_{i=1}^{n}\,{t}_{i}^{2}{\rm{tr}}[({\sum }_{m,h=1}^{{s}_{i}}\,{\beta }_{m}^{(i)}{\beta }_{h}^{(i)}{\hat{p}}_{m}^{(i)}{\hat{p}}_{h}^{(i)})\rho ]\\  & +\,\,{\sum }_{i\ne j}^{n}\,{t}_{i}{t}_{j}{\rm{tr}}[({\sum }_{m=1}^{{s}_{i}}{\sum }_{h=1}^{{s}_{j}}{\beta }_{m}^{(j)}{\beta }_{h}^{(j)}{\hat{p}}_{m}^{(j)}{\hat{p}}_{h}^{(j)})\rho ]\\ = & {\sum }_{i=1}^{n}\,{t}_{i}^{2}\,{\sum }_{m,h}^{{s}_{i}}\,{\alpha }_{m}^{(i)}{\alpha }_{h}^{(i)}{\rm{tr}}({\hat{x}}_{m}^{(i)}{\hat{x}}_{h}^{(i)}\rho )\\  & +\,\sum _{i\ne j}\,{t}_{i}{t}_{j}\,{\sum }_{m,h}^{{s}_{i},{s}_{j}}\,{\alpha }_{m}^{(j)}{\alpha }_{h}^{(j)}[{\rm{tr}}({\hat{x}}_{m}^{(j)}{\hat{x}}_{h}^{(j)}\rho )]\\  & +\,{\sum }_{i=1}^{n}\,{t}_{i}^{2}\,{\sum }_{m,h}^{{s}_{i}}\,{\beta }_{m}^{(i)}{\beta }_{h}^{(i)}{\rm{tr}}({\hat{p}}_{m}^{(i)}{\hat{p}}_{h}^{(i)}\rho )\\  & +\,\,\sum _{i\ne j}\,{t}_{i}{t}_{j}\,{\sum }_{m,h}^{{s}_{i},{s}_{j}}\,{\beta }_{m}^{(j)}{\beta }_{h}^{(j)}[{\rm{tr}}({\hat{p}}_{m}^{(j)}{\hat{p}}_{h}^{(j)}\rho )].\end{array}\end{array}$$

Now note that ${\hat{x}}_{m}^{(i)}={\hat{q}}_{2{s}_{1}+\cdots +2{s}_{i-1}+2m-1}$, ${\hat{p}}_{m}^{(i)}={\hat{q}}_{2{s}_{1}+\cdots +2{s}_{i-1}+2m}$, and *M*_*ρ*_ = (*m*_*ij*_) with ${m}_{ij}={\rm{t}}{\rm{r}}({\hat{q}}_{i}{\hat{q}}_{j}\rho ),{m}_{2k+1,2k+2}=$ ${\rm{t}}{\rm{r}}({\hat{q}}_{2k+1}{\hat{q}}_{2k+2}\rho )+\frac{i}{2},{m}_{2k+2,2k+1}={\rm{t}}{\rm{r}}({\hat{q}}_{2k+2}{\hat{q}}_{2k+1}\rho )-\frac{i}{2}$ we complete the proof.◽
